# Calixarene based portable sensor for the direct assay of indiscriminate ephedrine content of weight loss herbal preparations†

**DOI:** 10.1039/d0ra10254g

**Published:** 2021-04-06

**Authors:** Mary E. Wahba, Dalia El Wasseef, Ahmed S. Saad, Mohammed E. Draz

**Affiliations:** Department of Pharmaceutical Chemistry, Faculty of Pharmacy, Delta University for Science and Technology Gamasa Egypt; Department of Pharmaceutical Analytical Chemistry, Faculty of Pharmacy, Mansoura University Mansoura 35516 Egypt; Department of Medicinal Chemistry, Faculty of Pharmacy, Mansoura University Mansoura 35516 Egypt; Department of Analytical Chemistry, Faculty of Pharmacy, Cairo University 11562 Cairo Egypt ahmedss_pharm@yahoo.ocm ahmed.bayoumy@pharma.cu.edu.eg +20-1004009443; Pharmaceutical Chemistry Department, School of Pharmacy and Pharmaceutical Industries, Badr University in Cairo (BUC) Badr City 11829 Cairo Egypt

## Abstract

A novel potentiometric sensor was developed and optimized for the quantitative analysis of ephedrine in non-prescribed herbal supplements used as adjunctive therapy for weight loss. An initial optimization study aimed to reach the optimum membrane composition, sensor assembly, and experimental conditions. The study evaluated the effect of several factors on the sensor performance including different ion-exchangers, plasticizers, ionophores, membrane thicknesses, soaking solution concentrations, soaking time intervals, and pH. The optimized polyvinyl chloride membrane included tungstophosphoric acid hydrate as a cation exchanger, tricresyl phosphate as a plasticizer, and calix[8]arene as an ionophore to enhance the sensitivity and selectivity of the developed sensor. The polyvinyl chloride membrane was drop-casted over a polyaniline modified glassy carbon electrode surface to form a solid-state sensor. The proposed membrane succeeded to quantify ephedrine over a linear range of 6 × 10^−6^ to 1 × 10^−2^ M with a LOD of 3.60 × 10^−6^ M, acceptable selectivity, and fast response time. The IUPAC characterization of sensor response and International Conference on Harmonization validation parameters were calculated. The method successfully determined ephedrine concentration in spiked herbal mixtures and determined labeled and undeclared ephedrine content of weight loss herbal preparations.

## Abstract 2

Calixarene based solid-state potentiometric sensor for direct assay of indiscriminate ephedrine content in weight loss herbal products.

## Graphical abstract

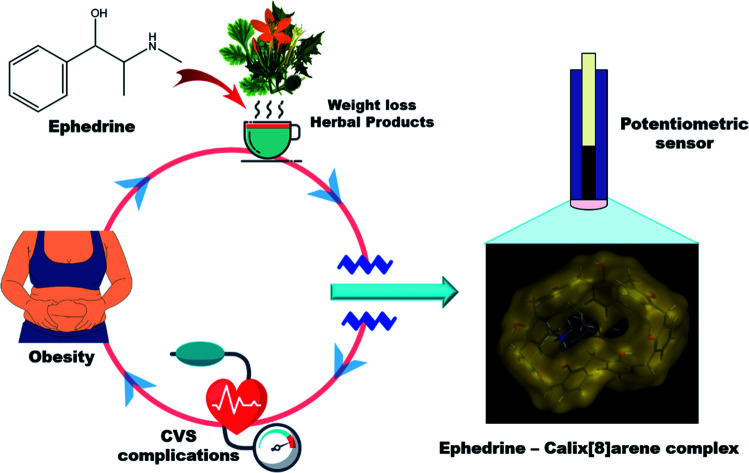

## 1. Introduction

Obesity is defined as the accumulation of body fat associated with a high body mass index. It is a predisposing factor for several health-threatening problems such as type 2 diabetes and cardiovascular disorders. Obesity treatment requires balanced management of the interrelation between food intake and the rate of metabolism to achieve the required body mass index.

Herbal remedies are classified as “dietary supplements” used as adjunctive therapy in dietary regimens.^1^ Unfortunately, the production of such botanical products is not overseen by most regulatory authorities such as the FDA.^2,3^ The latter neither necessitates safety and/or efficacy regulations before marketing nor surveys their adverse effects afterward. Weight loss herbal supplements include multi-component botanicals such as *Garcinia cambogia*, Indian bdellium, *Terminalia chebula*, green coffee bean, *etc.* that exert stimulant, diuretic, and/or metabolic effects.^3^ Synergistic effects of such components in a single formulation might enhance the encountered side effects.^3^

**Fig. 1:**
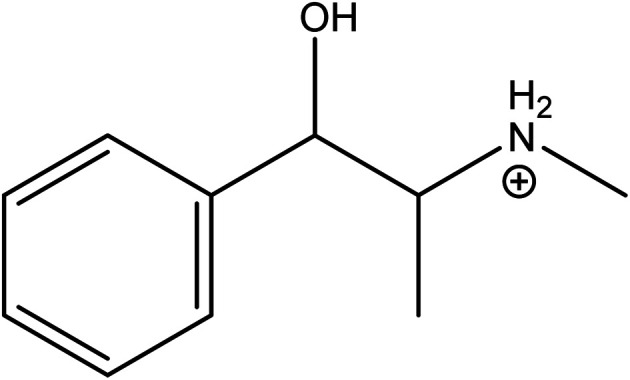
Chemical structure of ephedrine.

Ephedrine (EPH) (Fig. 1) is the main alkaloid in the Chinese herb Ma Huang.^4^ It is included in many natural herbal products designed for weight loss. EPH depresses the appetite, especially when combined with caffeine.^3^ The sympathomimetic behavior of EPH arises from its action at the receptor sites after releasing nor-epinephrine to the extracellular fluid.^4^ It exerts an adrenergic effect as an α1, α2, β1, and β2 receptor agonist as well as the release of endogenous norepinephrine.^4^ Consequently, it exhibits pronounced side effects including hypertension, tachycardia, and in severe cases it causes strokes that may eventually lead to death.

There seems to be a need for a reliable analytical method for EPH assay in marketed weight loss formulations. Several research articles in the literature were devoted to EPH assay, where liquid chromatography was the predominantly applied analysis tool,^5–11^ in addition to gas chromatography,^12^ and electrophoresis.^13^ The relatively high polarity of EPH necessitated the use of either chromatographic columns with special chemistries or the addition of mobile phase modifiers to retain EPH within the stationary phase and thereby enable the efficient chromatographic separation process. Furthermore, EPH was assayed in different pharmaceutical formulations and biological fluids using PVC potentiometric sensors^14–20^ but neither of them succeeds to determine EPH in herbal complex mixtures. Moreover, they relied on traditional liquid membranes with an inner filling solution that promote the transmembrane flux rather than the solid contact sensors which showed better sensitivities.^21^

The development of a reliable, sensitive, and selective analytical method for EPH assay has challenged analysts for a long time. The reported analytical methods included complicated extraction procedures of the herbal mixtures such as boiling with methanol^7^ or refluxing in a water bath for several hours.^9^ Ephedrine chemical structure shows a weakly conjugated chromophore represented in a single benzene ring which exhibits a low sensitivity and non-selective light absorbance. Analysts had to sacrifice sensitivity for selectivity or *vice versa*. Therefore, high sensitivity determinations require either derivatization procedures or special detectors. Ephedrine's hydrophilic and basic nature throughout the allowable chromatographic pH range (pH 2–8), hinders its retention and causes peak broadening and tailing on traditional reversed-phase analytical columns. Consequently, former chromatographic assays employed special stationary phases (*e.g.* pentafluorophenyl column)^5^ or ion-pairing agents.^8–10^ The latter suppresses ionization^5^ and impedes the utility of mass detection which renders the identification of EPH among other multi-component herbal remedies challenging.

Electrochemical sensors provide a tool for the selective and direct assay of chemicals in complex matrices. They are the methods of choice in turbid and colored samples and wherever sample preparation is undesirable. Potentiometric ion-selective electrodes are the type of electrochemical sensors that measures the potential difference established at the interface between the sample and the membrane selective to the analyte ion. Potentiometric sensors introduce sustainable, portable, direct, and real-time analytical solutions for the assay of ionizable analytes in complex sampling matrices. Sustainable potentiometric methods avoid sample preparation and derivatization to minimize waste production and chemicals, energy, time, and laboratory-resources consumption.^22^

The current work optimizes and develops a solid-state potentiometric sensor for EPH assay in dietary supplements that claim to manage obesity. The sensor was based on a poly(vinyl) chloride (PVC) membrane drop-casted over polyaniline (PANI) coated glassy carbon electrode. The study optimized the composition of the PVC membrane for maximum sensitivity and selectivity for EPH. During the study, the method was designed to abide by the concepts and regulations of green analysis to develop an eco-friendly analytical method that consumes and produces the least resources and effluents per analysis.

## 2. Experimental

### 2.1. Instrument

Potentiometric measurements were performed using Jenway (Model 3505) pH/mV meter (Dunmow, Essex, England). An external reference electrode (double junction Ag/AgCl) was utilized; Thermo scientific, Orion, 900200.

CHI 104 Glassy Carbon Disk Working Electrode, imported from CH Instruments, Inc., Tennison Hill Drive, Austin, USA.

The membrane thickness was measured using a digital caliper (Model: GMC-190, Taiwan).

### 2.2. Materials and reagents

Ephedrine sulphate (EPH) was provided by (Chemical Industries Development CID, Cairo, Egypt). Tetrahydrofuran (THF) was purchased from (Scharlau, the Lab Sourcing Group, Barcelona, Spain). High molecular weight poly(vinyl) chloride (PVC), dibutyl phthalate (DBP), dioctyl phthalate (DOP), calix[4]arene-25,26,27,28-tetrol (CX-4), calix[6]arene (CX-6), calix[8]arene (CX-8), aniline were all purchased from (Sigma Aldrich, Missouri, USA). Sodium tetraphenylborate of purity 99% (TPB), 12-molybdophosphoric acid (PM), tricresyl phosphate (TCP), 2-nitrophenyl phenyl ether (NPPE), 2-nitrophenyl octyl ether (NPOE), and ammonium reineckate (AR) were all purchased from (Alfa Aesar, Massachusetts, USA). Tungstophosphoric acid hydrate (PT) was purchased from (Merck, Darmstadt, Germany). β-Cyclodextrin (β-CD) was purchased from (Acros Organics, New Jersey, USA). Potassium dihydrogen phosphate, prepared as 50 mM in distilled water, hydrochloric acid, and sodium hydroxide were purchased from (El-Nasr Pharmaceutical Chemical Company, Cairo, Egypt). Royal Regime Tea® bags, each tea bag contains 20% *Cichorium intybus* herb, 30% *Cassia angustifolia* leaves, and 50% *Foeniculum vulgare* fruits, batch # 12937, manufactured by Ottoman-Cairo ARE (Royal Herbs), Egypt. A herbal mixture of unknown composition purchased from a local apothecary.

### 2.3. Preparation of standard solutions

The stock solution of EPH was prepared as 1.00 × 10^−2^ M by dissolving in 50 mM KH_2_PO_4_ buffer and the final solution was adjusted to pH 5.5 using phosphoric acid. Appropriate dilutions in the range 2.00 × 10^−6^ to 1.00 × 10^−2^ M were made by dilution of the stock solution using the same buffer.

### 2.4. Procedures

#### 2.4.1. Polymeric membrane preparation

Membrane cocktails for sensors 1 through 8 were separately prepared in a 5 mL volumetric flask by mixing accurately weighed amounts of the membrane components; PVC, plasticizer (TCP, DBS, NPPE, NPOE, DOP or NPOE), and ion-exchanger (TPB, PT, PM, or AR) (Table 1), while keeping the ratio of PVC: plasticizer: ion exchanger at 31.66 : 66.66 : 1.67%, w/w. Membrane cocktails for sensors 9 through 13 included an additional accurately weighed amount of an ionophore CX-4, CX-6, CX-8, β-CD, and β-CD : CX (1 : 1), respectively (Table 2). The components were mixed, dissolved in THF and the volume was completed to the mark using THF.

**Table:** Effect of the PVC membrane composition on the performance characteristics of the prepared sensors^b^

Sensor	Composition		Performance		
	Plasticizer	Ion exchanger	Linearity range (M)	Slope^a^ (mV per decade)	*r* a
1	TCP	TPB	1.58 × 10^−5^ to 3.85 × 10^−3^	34.92	0.9713
2	TCP	PM	1.58 × 10^−5^ to 3.85 × 10^−3^	30.96	0.9907
3	TCP	AR	1.58 × 10^−5^ to 3.85 × 10^−3^	49.83	0.9879
4	TCP	PT	1.58 × 10^−5^ to 1.00 × 10^−2^	57.67	0.9965
5	DBP	PT	4.75 × 10^−5^ to 3.85 × 10^−3^	35.42	0.9793
6	DOP	PT	5.87 × 10^−7^ to 1.28 × 10^−3^	15.95	0.9918
7	NPPE	PT	5.87 × 10^−7^ to 4.28 × 10^−4^	10.56	0.9921
8	NPOE	PT	1.96 × 10^−7^ to 3.85 × 10^−3^	4.34	0.9339

a Average of three determinations.

b Tricresyl phosphate (TCP), tungstophosphoric acid hydrate (PT), sodium tetraphenyl borate (TPB), 12-molybdophosphoric acid (PM), ammonium reineckate (AR), dibutyl phthalate (DBP), dioctyl phthalate (DOP), 2-nitrophenyl phenyl ether (NPPE), 2-nitrophenyl octyl ether (NPOE).

**Table:** The calculated *P* values using one-way ANOVA statistical analysis of the results obtained from the optimization study

Parameter	*P* value					
	Ion exchanger	Plasticizer	Ionophore	Conditioning time	Conditioning solution concentration	Membrane thickness
Slope	<0.0001	<0.0001	<0.0001	**0.8058**	<0.0001	<0.0001
LOQ	NA	<0.0001	<0.0001	**0.6739**	<0.0001	<0.0001
*r*	<0.0001	<0.0001	0.0004	**0.3531**	<0.0001	<0.0001

#### 2.4.2. Sensor assembly

A slurry anchored on Al_2_O_3_ was sonicated for 15 minutes to gloss the surface of the glassy carbon electrode then rinsed with acetone to finish the polishing phase. The electropolymerization of PANI on the glassy carbon surface was developed by cyclic voltammetry using three electrodes chemical cell containing 1 M HCl and 0.45 M aniline. The potential was rounded between −0.2 V and 0.8 V in 2 mV steps at a 50 mV s^−1^ scan rate for 5 consecutive cycles to complete coating of glassy carbon electrode with PANI. An accurate volume of the cocktail was drop casted onto the polished surface of the PANI coated glassy carbon electrode and left to dry for 30 minutes to obtain the membrane sensors. The prepared sensors were preconditioned by soaking in a 1.00 × 10^−2^ M EPH solution for 1 h before carrying out the potentiometric measurements.

#### 2.4.3. Sensor calibration

The pre-conditioned sensors in conjunction with the reference electrode were immersed in EPH standard working solutions over the range of 2.00 × 10^−6^ to 1.00 × 10^−2^ M. The potential difference between the two electrodes (mV) was recorded while stirring at 200 rpm. The sensors were washed with distilled water between measurements. The calibration curves were then constructed by plotting the obtained potential values (mV) *versus* the logarithmic concentrations of EPH. Eventually, regression equations were deduced.

#### 2.4.4. Validation of the sensor performance

The IUPAC recommendations^23^ were followed to calculate the sensor performance parameters such as LOD, linear range, dynamic response time, and reversibility of the sensor response (ESI S1†).

Eventually, the International Conference on Harmonization (ICH)^24^ validation parameters were evaluated to characterize the performance of the optimized sensor.

#### 2.4.5. Sensor selectivity

The separate solution method recommended by the IUPAC^25^ was adopted to compute the potentiometric selectivity coefficient (*K*^pot^_EPH,I_) to characterize the sensor response to commonly interfering ions: K^+^, Na^+^, NH_4_^+^, Ca^2+^, Ni^2+^, Mg^2+^, Mn^2+^, Co^2+^, Cu^2+^, Cd^2+^, Pb^2+^ and Fe^3+^.1
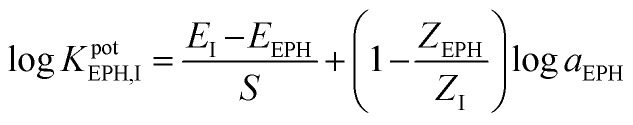


The selectivity coefficient is expressed as log*K*^pot^_EPH,I_, *E*, *S*, and *Z* refer to the measured potential, sensor slope (for EPH), ionic charge, respectively, while the subscript I and EPH denote the interfering ions and EPH, respectively. Since the effect of ionic strength is minimal and activity coefficient approaches unity at the relatively low concentrations at which measurements were carried out, the molar concentration was used instead of the activity (*a*).

#### 2.4.6. Effect of pH

The influence of pH changes on the potential of the constructed electrode was studied in each of 1 × 10^−3^ and 1 × 10^−4^ M ephedrine solutions separately, over the pH range 2–12. The potential and pH were simultaneously measured in the studied EPH solution after incremental addition of minute volumes of either 1 M HCl or 1 M NaOH. The potential measurements were carried out using the optimized sensor in conjugation to the double junction Ag/AgCl reference electrode. The pH was simultaneously measured in the same solution using a glass electrode. Eventually, the resultant potential readings were plotted *versus* pH for each EPH concentration.

#### 2.4.7. Application to herbal dietary supplements

Standard addition methodology^26^ was utilized during this application. Herbal remedies were prepared as an infusion to simulate their domestic routine preparation steps by the consumers. The content of five tea bags of the herbal tea (Royal Regime Tea®) or an equivalent amount of herbal mixture of unknown composition was weighed and the weight (*w*) was recorded. The content was mixed in a mortar, and an amount equivalent to the content of one tea bag or 15 g were transferred to a glass stoppered conical flask, 200 mL of boiling water were added, the flask was stoppered and left for 10 minutes while stirring at 200 rpm. The extract was left to cool to room temperature, and then 25 mL were transferred to a beaker. The potential was recorded before and after the addition of 1 mL 10^−2^ M EPH solution. EPH intake per serving and concentration in the herbal products were calculated by applying the following equation:^27^2
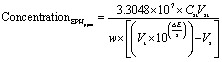


The concentration (ppm) of EPH (concentration_EPH_ppm__) in the herbal tea powder was calculated. The change in potential (Δ*E*) was recorded after the addition of milliliters of the standard (*V*_st_) of known molar concentration (*C*_st_) to the sample solution of volume (*V*_s_) measured in milliliters, whereas (*V*_t_) is the total milliliters of the two solutions (*V*_s_ + *V*_st_), (*s*) is the slope in mV per decade of the sensor response to EPH calculated from a recent calibration, and (*W*) is the average mass in grams of a single serving of a herbal tea packet or herbal mixture. Substitution in eqn (2) the concentration (ppm) of ephedrine base in the herbal tea powder or herbal mixture. The derivation of the equation is represented in ESI S2.†

## 3. Results and discussion

Potentiometric sensors based on PVC have idiosyncratic characteristics. PVC forms a microscopic molecular network through which the plasticizer enables the free movement of the ionic species within the membrane matrix to enhance membrane conductance,^28^ these unique electric features of PVC membranes were thoroughly investigated in literature.^27–31^ The rapid response and high selectivity of PVC-based potentiometric sensors are attributed to the presence of dissociated anchored sites for ion exchange, host–guest supramolecular chemical interaction, and complex formation within the internal sensor matrix.^28–30^ Therefore, the PVC matrix has proven to be a felicitous candidate in the potentiometric sensor industry.

The optimization study was carried out to achieve the desired sensor performance. The study investigated the type and amount of the components incorporated within the PVC membrane. The study evaluated factors related to sensor assembly (*e.g.* sensor composition, membrane thickness) and experimental conditions (*e.g.* soaking solution concentration and soaking time, pH). The optimization aimed to reach sensor composition and conditions that guarantee optimal sensor performance.

Ephedrine has a basic nature (basic p*K*_a_ = 10.3) and predominantly behaves as a monovalent cation at a pH lower than 8.3.^32^ Therefore, preliminary measurements were carried out at a slightly acidic pH (pH = 5.5) at which EPH carries a single positive charge over its amino group as shown in (Fig. 1).

We analyzed the results of the optimization study statistically using one-way ANOVA tests. Each ANOVA test compares the effect of different levels of a single factor, on a single performance parameter (slope, LOQ, and correlation coefficient). The statistical analysis reveals significant differences between the studied levels (Table 2).

The statistical analyses of the performance results revealed that except for the conditioning time and ion-exchanger, different levels of the studied factors engender significantly different slopes, LOQs, and correlation coefficients. The studied levels of the ion-exchanger resulted in a significantly different slope and correlation coefficient but did affect the LOQ (Table 2). A significant difference among a factor necessitates careful selection of the working level used for sensor fabrication. Thus, we performed *post hoc* analyses within each factor to explore differences among its levels.

**Fig. 2:**
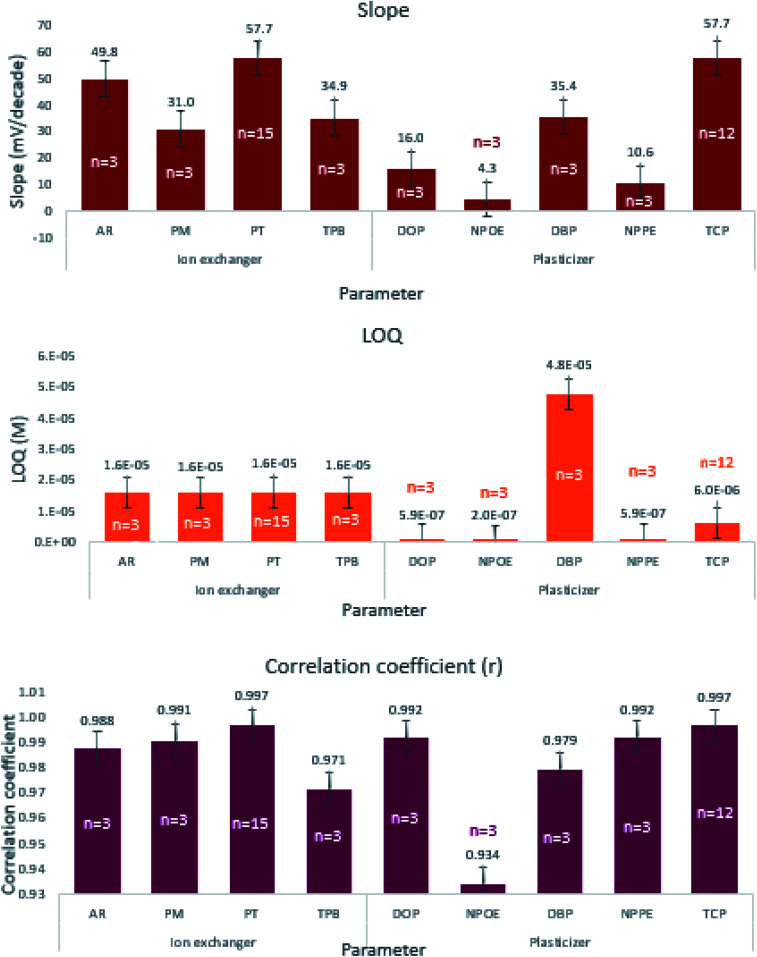
Effect of different ion-exchangers and plasticizers on the slope, LOQ, and correlation coefficient of the prepared sensors. Where, tricresyl phosphate (TCP), tungstophosphoric acid hydrate (PT), sodium tetraphenylborate (TPB), 12-molybdophosphoric acid (PM), ammonium reineckate (AR), dibutyl phthalate (DBP), dioctyl phthalate (DOP), 2-nitrophenyl phenyl ether (NPPE), 2-nitrophenyl octyl ether (NPOE), N.B. vertical lines represent standard error bars.

We selected the working levels of each component based on their ability to engender the desired performance, namely the nearest Nernstian slope, minimum LOQ, and maximum correlation coefficient. We studied the effect of four different cation exchangers, namely PT, TPB, PM, and AR sensors 1 through 4 while using TCP as a plasticizer (Table 1). The higher slope indicates faster exchange kinetics of EPH through the formation of a stable ion-pair complex. One way ANOVA statistical analysis revealed significant differences among the resulting slopes and correlation coefficients (Table 2). Fig. 2 shows that sensor-4 containing PT achieved the highest correlation coefficient (0.9965) and the best Nernstian slope (57.67 mV per decade) than any of the other three sensors. However, the *post hoc* statistical analysis revealed that sensor-4 slope and correlation coefficient did not significantly differ from sensor-3 containing AR, but both differ significantly from sensors 1 and 2 containing TPB and PM (ESI Table S3a†).

Plasticizers are embedded between the polymer chains, spacing them apart, causing an increase in the free membrane volume. They act as solvents that mediate the movement of ionic species within the membrane matrix.^33,34^ Plasticizers are the most abundant membrane component that represents around two-thirds of the membrane composition to guarantee mobility within the PVC matrix and to enhance membrane flexibility and elasticity. The utilized plasticizer should be compatible with PVC, possessing high lipophilicity and low viscosity.^33,34^

The study investigated the influence of five different plasticizers namely DBP, DOP, NPPE, NPOE, and TCP, while using PT as the ion-exchanger throughout sensors 4 through 8 (Table 1). Sensor-4 containing TCP demonstrated adequate Nernstian response, wide linear range, low LOQ, and high correlation coefficient (Fig. 2). One way ANOVA proved significant differences among the studied plasticizer levels on the slope, LOQ, and correlation coefficient (Table 2). The *post hoc* analysis of the five plasticizer levels proved that TCP possessed a significantly different slope from the four plasticizers. Sensors 4, 6, 7, and 8 containing TCP, DOP, NPPE, and NPOE resulted in low quantification limits that did not differ from each other but significantly different from the relatively higher LOQ obtained using DBP. TCP expressed the highest correlation coefficient which differs significantly from that of DBP and NPOE (ESI Table S3b†). The low dielectric constant TCP engendered the appropriate membrane polarity to extract the monovalent EPH, thus fulfilled the Nernstian membrane behavior.^33^

Ionophores are complexing agents with lipophilic nature.^35^ The high lipophilicity guarantees strong retention within the membrane to increase the stability of the response and the lifetime of the sensor. Ion-selective electrodes containing ionophores exhibit different selectivity patterns than ionophore-free sensors.^36^ The ionophore chemical structure includes polar functional groups embedded within the vicinity of their lipophilic structure. These polar functional groups play a vital role to recognize the analyte ions.^36^ These differences arise from the formation of a stable complex between the ionophore and the analyte ion within the membrane matrix.^36^ The selectivity of membranes based mainly on ion exchangers depends on the solvation energy of the analyte ions. On the other hand, the selectivity of ionophore-based membranes includes factors such as the ions' free energy of transfer from the aqueous phase to the membrane, ability of ionophore to extract the ions selectively which is considered more influential than the binding strength of the formed complex.^35^

**Fig. 3:**
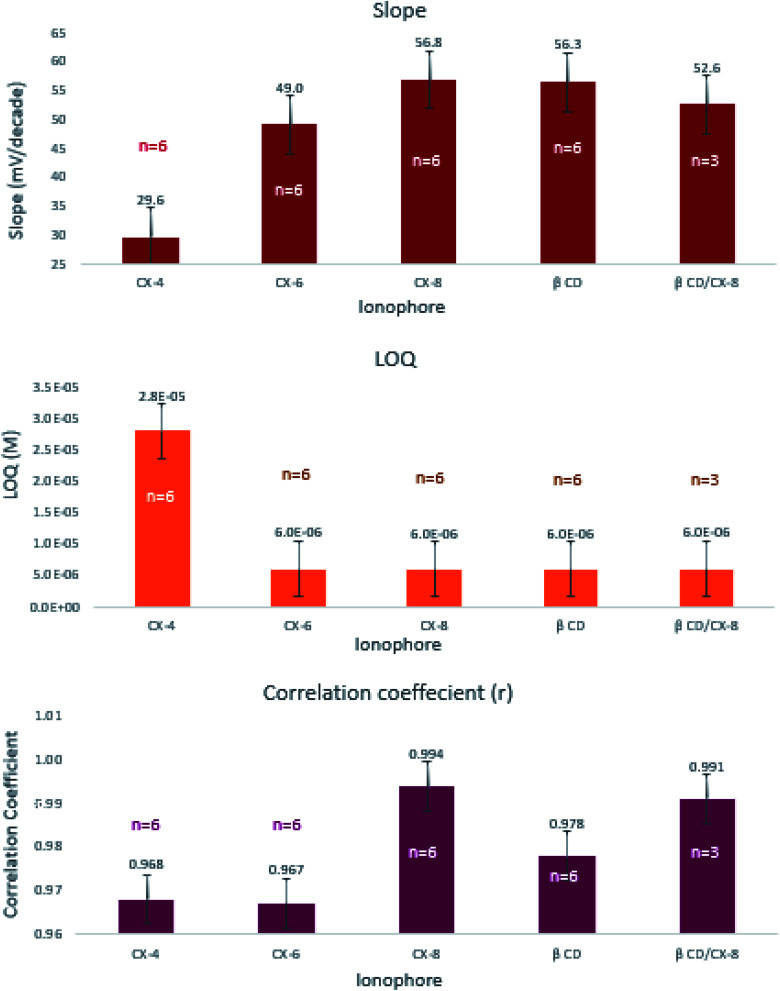
Effect of different ionophores on the slope, LOQ, and correlation coefficient of the prepared sensors. Where, calix[4]arene-25,26,27,28-tetrol (CX-4), calix[6]arene (CX-6), calix[8]arene (CX-8), β-cyclodextrin (β-CD), N.B. vertical lines represent standard error bars.

We studied the ability of four different ionophores (CX-4, CX-6, CX-8, and β-CD) and an ionophore mixture (CX-8 : β-CD, 1 : 1) to enhance the performance characteristics of the sensor containing PT as an ion-exchanger and TCP as a plasticizer (Table 3). The ionophores were incorporated in proportion double that of PT. Visual inspection of the results shows that sensor-11 containing CX-8 had the best Nernstian behavior, wide linear range, high correlation coefficient, and fast response time (Fig. 3). Except for CX-4, the slopes and quantification limits obtained using the other four ionophores did not differ significantly from one another. The highest correlation coefficients were obtained using CX-8 and CX-8/β-CD mixture which statistically differ from those obtained using any of the other ionophore levels (ESI Table S3c†).

**Table:** Effect of ionophore types and conditioning time on the performance of the prepared sensors^b^

Sensor	Parameter		Performance		
	Ionophore	Conditioning time	Linearity range (M)	Slope^a^ (mV per decade)	*r* a
9	CX-4	One hour	3.79 × 10^−5^ to 1.00 × 10^−2^	27.46	0.9549
	CX-4	Overnight	1.80 × 10^−5^ to 1.00 × 10^−2^	31.77	0.9810
10	CX-6	One hour	6.00 × 10^−6^ to 1.00 × 10^−2^	44.62	0.9539
	CX-6	Overnight	6.00×10^−6^ to 2.86 × 10^−3^	53.43	0.9802
11	CX-8	One hour	6.00 × 10^−6^ to 1.00 × 10^−2^	58.86	0.9985
	CX-8	Overnight	6.00 × 10^−6^ to 1.00 × 10^−2^	56.59	0.9903
12	β-CD	One hour	6.00×10^−6^ to 1.00 × 10^−2^	56.34	0.9778
	β-CD	Overnight	6.00 × 10^−6^ to 1.00 × 10^−2^	58.69	0.9919
13	CX-8 : β-CD (1 : 1)	One hour	6.00 × 10^−6^ to 1.00 × 10^−2^	52.56	0.9910

a Average of three determinations.

b Calix[4]arene-25,26,27,28-tetrol (CX-4), calix[6]arene (CX-6), calix[8]arene (CX-8), β-cyclodextrin (β-CD).

**Fig. 4:**
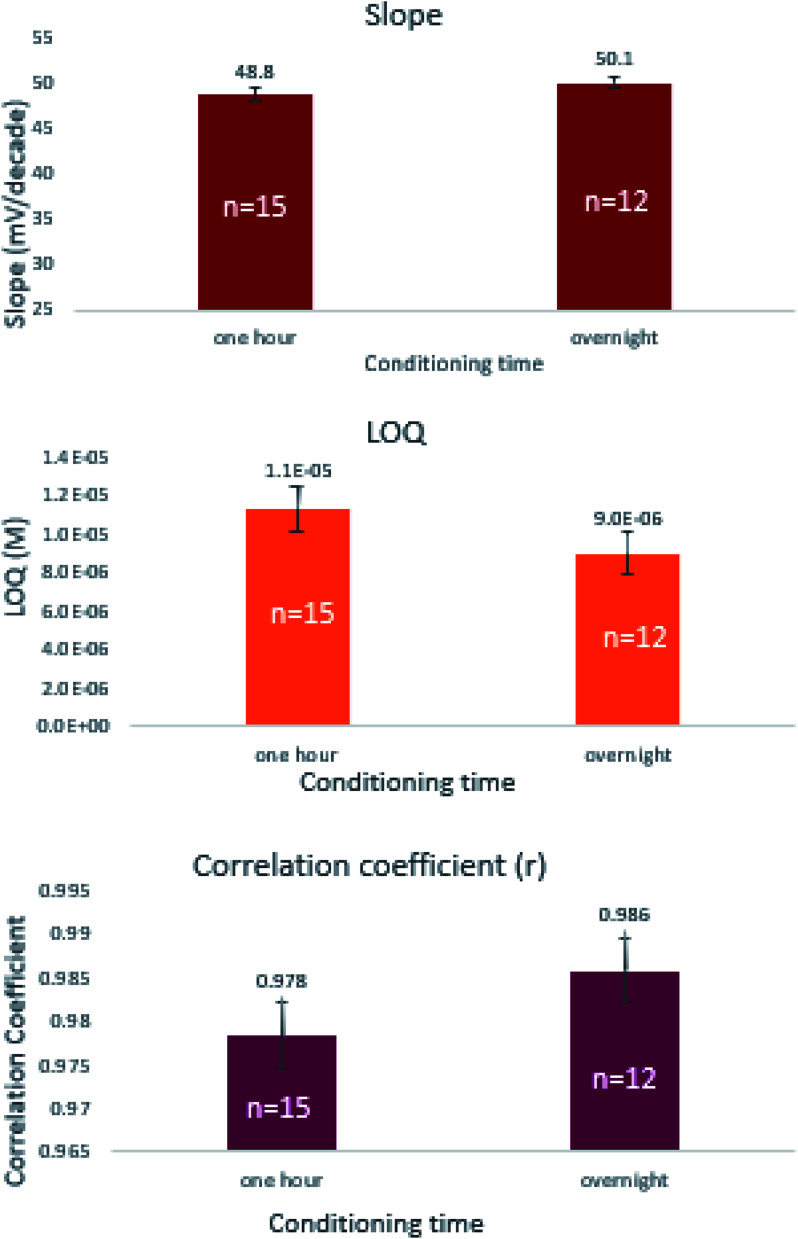
Effect of soaking time on the slope, LOQ, and correlation coefficient of the prepared sensors, N.B. vertical lines represent standard error bars.

**Fig. 5:**
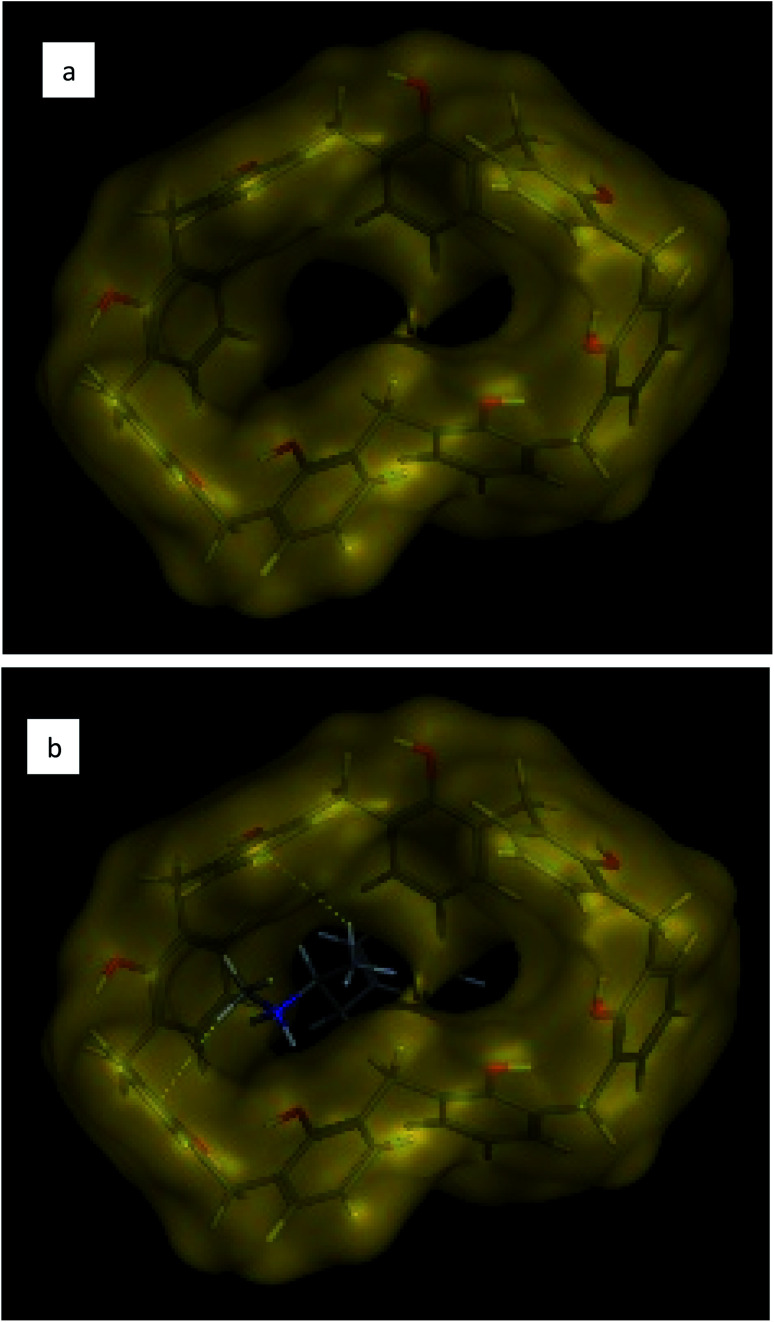
Three-dimensional chemical structure of (a) calix[8]arene and (b) hydrophobic interaction of calix[8]arene with ephedrine.

The effect of soaking time was studied at one hour and 24 hours' time intervals. Results revealed insignificant differences in the measured responses (Fig. 4). Comparing the slopes of sensors 9 through 12, sensor-11 expressed an exceptionally fast conditioning time and reached a stable Nernstian slope and high correlation coefficient after 1 hour (Table 3). This emphasizes that CX-8 forms a sterically and energetically more favored inclusion complex with EPH than any of the other ionophores. The electronegative carbonyl groups of CX-8 bind the protonated amino group of EPH *via* hydrogen bonds, to coincide the hydrophobic moiety of EPH with the lipophilic part of CX-8^37^ (Fig. 5).

Furthermore, different concentrations of EPH were used for soaking (1.00 × 10^−2^, 1.00 × 10^−3^, and 1.00 × 10^−4^ M). Better Nernstian slope, wider linear range, and higher correlation coefficient were achieved using 1.00 × 10^−2^ M. The *post hoc* statistical analysis showed that the three concentration levels expressed significantly different slopes, LOQs, and the correlation coefficients from each other (ESI Table S3d†). The high concentration of the relatively polar EPH molecule was mandatory to reach equilibrium at the sensor–sample interface, while lower concentrations produced unacceptable Nernstian behavior (Table 4), since 1.00 × 10^−2^ M is expected to provide sufficient activity for the ion exchange process.

Effect of the conditioning solution concentration and membrane thickness on the performance of the developed sensor

**Table:** 

Effect of concentration of EPH solution for conditioning			
Concentration of EPH conditioning solution (M)	Linearity range (M)	Slope^a^ (mV per decade)	*r* a
1.00 × 10^−2^	6.00 × 10^−6^ to 1.00 × 10^−2^	58.86	0.9985
1.00 × 10^−3^	6.90 × 10^−6^ to 1.00 × 10^−3^	44.83	0.9916
1.00 × 10^−4^	6.90×10^−6^ to 1.00 × 10^−3^	52.25	0.9897

a Average of three determinations.

**Table:** 

Effect of membrane thickness			
Thickness	Linearity range (M)	Slope^a^ (mV per decade)	*r* a
0.12 mm	6.00 × 10^−6^ to 1.00 × 10^−2^	55.96	0.9988
0.10 mm	6.00 × 10^−6^ to 1.00 × 10^−2^	58.86	0.9985
0.08 mm	6.00 × 10^−6^ to 1.00 × 10^−2^	62.47	0.9988

The PVC membrane behaves as an ionic conductor due to the mobilities of ions. The resistance of these conductors is a function of the thickness of membrane and mobilities of ions^38^ so the influence of membrane thickness on sensor performance was investigated at three different levels, namely 0.08, 0.1, and 0.12 mm (Table 4). The *post hoc* analysis expressed significant differences among the performance parameters (slopes, quantification limits, and correlation coefficients) obtained using the three thickness levels (ESI Table S3e†). The nearest Nernstian slopes were achieved using 0.1 mm thick membranes.

**Fig. 6:**
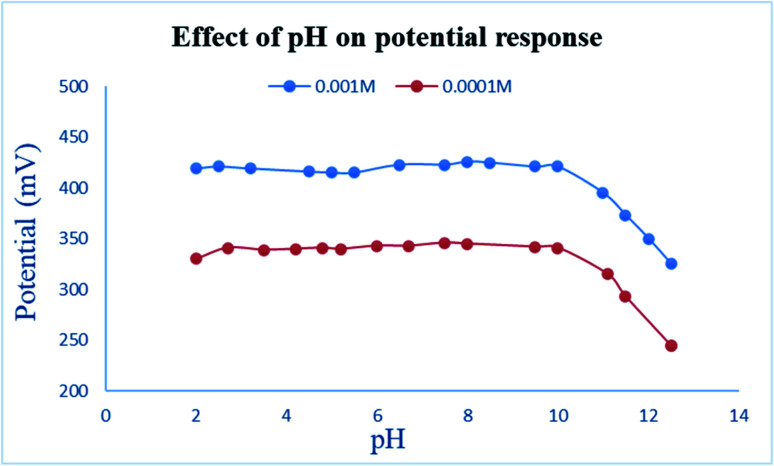
Effect of pH on the measured potential of 1.00 × 10^−3^ and 1.00 × 10^−4^ M EPH solutions using the developed sensor.

The pH study revealed a constant potential profile in the pH range 2 through 10 over two different concentrations of EPH (10^−2^ and 10^−3^ M) (Fig. 6). Beyond pH 10 the potential sharply dropped due to deprotonation of EPH to lose its cationic nature, resulting in the unionized ephedrine base. These results are in accordance with the basic nature of EPH that possesses a basic p*K*_a_ value of 10.3.^32^

**Fig. 7:**
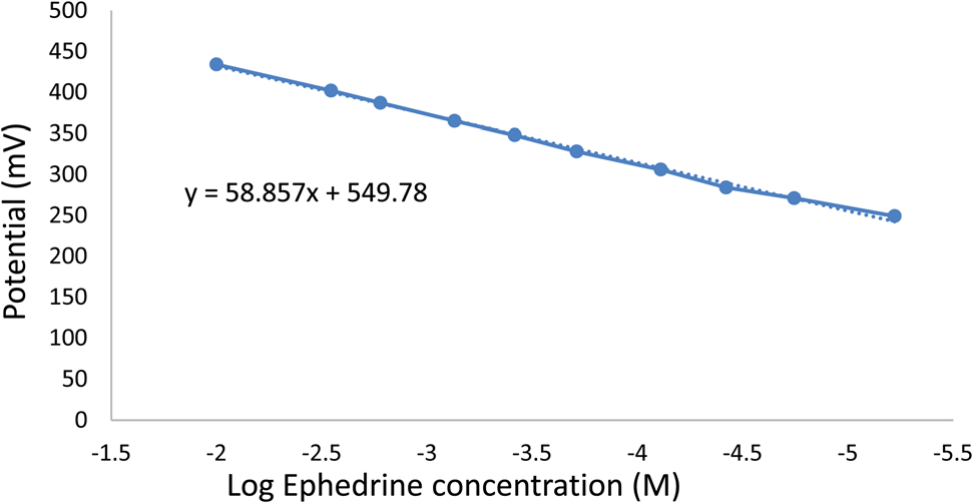
Calibration curve for EPH using a sensor containing PVC (31.66%), PT (1.6%), TCP (66.66%), and modified with CX-8, N.B. vertical lines represent standard error bars.

Optimum sensor performance was obtained using a 0.1 mm membrane thick containing PT as ion-exchanger, TCP as a plasticizer, and CX-8 as ionophore. The sensor was preconditioned in 1.00 × 10^−2^ M EPH solution for one hour before measurements. The optimized sensor demonstrated a superior fast linear potential response for the logarithm of the molar concentration of EPH with a slope of 58.86 ± 2 mV per decade in the concentration range 6.00 × 10^−6^ to 1.00 × 10^−2^ M (Fig. 7).

The calculated selectivity coefficients in Table 5, proved a relatively higher sensor selectivity towards EPH than any of the studied interferents. The incorporation of CX-8 as an ionophore enhanced the membrane selectivity for EPH ions through selective extraction of EPH ions from the aqueous sample solution rather than other interfering ions.^35^

**Table:** Selectivity coefficient *K*^pot^_EPH,I_ of the constructed electrode towards interfering ions

Interfering ions	Selectivity coefficient^a^*K*^pot^_EPH,I_
K^+^	3.54 × 10^−4^
Na^+^	3.84 × 10^−4^
Ca^2+^	1.27 × 10^−3^
Pb^2+^	1.30 × 10^−3^
Cd^2+^	4.10 × 10^−4^
Ni^2+^	1.40 × 10^−3^
Mg^2+^	1.10 × 10^−3^
Mn^2+^	3.20 × 10^−4^
Fe^3+^	1.70 × 10^−3^
Cu^2+^	3.80 × 10^−4^
NH_4_^+^	1.60 × 10^−3^
Co^2+^	3.54 × 10^−4^

a Average of three determinations.

**Fig. 8:**
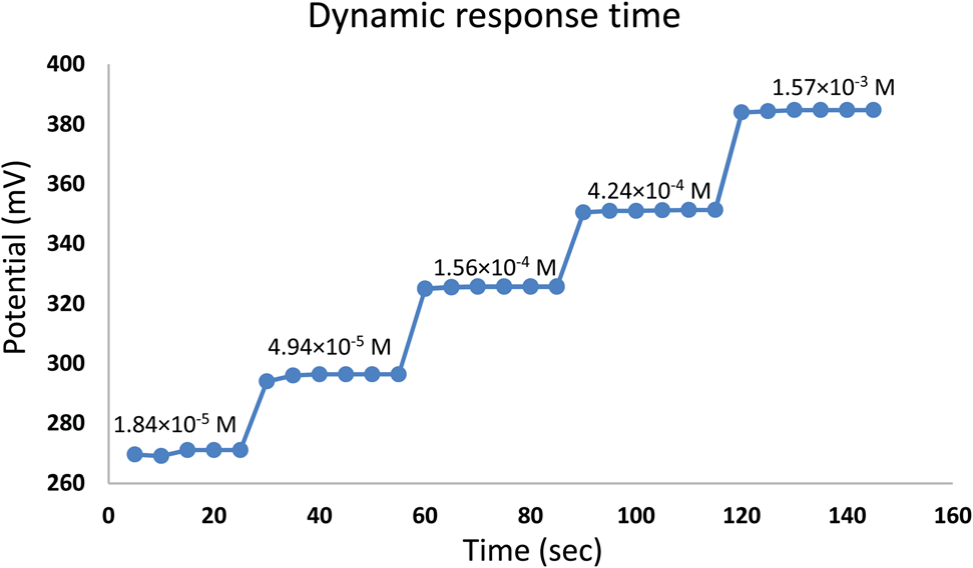
Dynamic response time curve of the developed sensor.

**Fig. 9:**
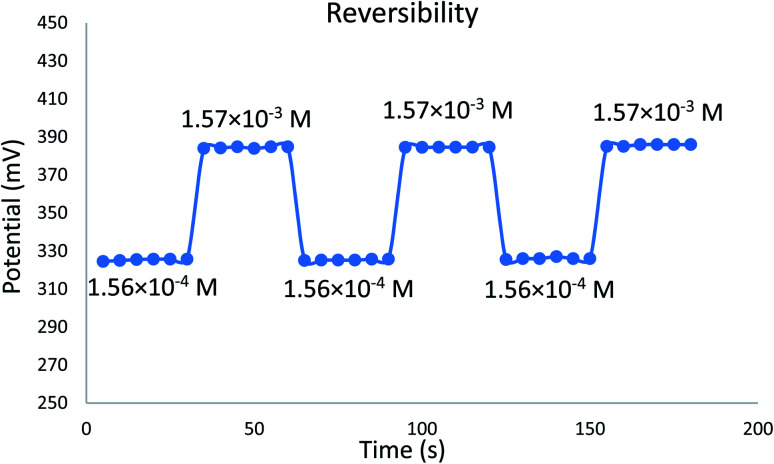
Potential reversibility of the developed sensor measured in 1.56 × 10^−4^ and 1.57 × 10^−3^ EPH solutions.

The time required for the sensor to reach equilibrium potential ±1 mV was studied while varying the concentration of EPH (1.84 × 10^−5^ to 1.57 × 10^−3^ M). The sensor expressed an average dynamic response time of 6 ± 1 seconds (Fig. 8). The sensor expressed a relatively fast reversibility upon oscillation between two different concentrations of EPH (1.56 × 10^−4^ and 1.57 × 10^−3^ M). The relatively fast exchange kinetics proved the ability of the sensor to equilibrate EPH ions rapidly on its surface (Fig. 9).

To address the sensor life-time, calibrations were frequently performed (weekly) using the optimized sensor and the slope was calculated. Meantime the sensor was stored in a 1.00 × 10^−2^ M EPH solution. The sensor proved a stable Nernstian slope ±2 mV per decade for four weeks (Table 5).

ICH guidelines^24^ were adopted to validate the proposed potentiometric method. Various concentrations of EPH standard solutions were analyzed thrice to assess the linearity. A good correlation coefficient was obtained to indicate good linearity as shown in (Table 6). The accuracy of the proposed method was evaluated over three different concentrations covering the span of the linear range (1.00 × 10^−2^, 7.00 × 10^−5^, 6.00 × 10^−6^ M) and recovery percentages were deduced from the corresponding regression equation. Results confirmed the accuracy of the method (Table 6).

**Table:** Electrochemical response characteristics of the proposed sensor

Parameter		Response^a^
Concentration range (M)		1.00 × 10^−2^ to 6.00 × 10^−6^
Linearity	Slope (mV per decade)	58.86
	Intercept (mV)	549.78
	Correlation coefficient	0.9985
Accuracy (mean ± SD)		101.59 ± 0.971
Precision	Repeatability^b^ (% RSD)	±1.574
	Intermediate precision^c^ (% RSD)	±2.119
LOD^d^ (M)		3.60 × 10^−6^ M
Response time (s)		8
Working pH range		2–10
Stability (days)		30

a Average of five determinations.

b The intraday (*n* = 3), an average of three different concentrations repeated three times within the day.

c The interday (*n* = 3), an average of three different concentrations repeated three times in three successive days.

d Limit of detection calculated according to the IUPAC recommendations.

The previous concentrations were analyzed three times within the day and on three consecutive days to estimate the repeatability intermediate precision, results proved the precision of the potentiometric method (Table 6).

As suggested by the IUPAC guidelines,^23^ the limit of detection (LOD) was determined at the intersection point of the linear extrapolated part of the calibration curve. The proposed sensor exhibited a relatively low detection limit (Table 6).

Selectivity, sensitivity, precision, and rapid response nominates the optimized sensor for EPH assay in herbal remedies without previous sample preparation or separation in contrast to the reported methods. The standard addition technique was adopted to determine EPH to deter the undesirable effect of variable ionic strength of the sample solution which alters EPH activity and thereby the measured potential.

It was noticed that herbal mixtures from known sources like Royal Regime Tea® do not include EPH. Unfortunately, results indicate that herbal mixtures from the local apothecary contained a considerable amount of EPH (Table 7).

**Table:** Determination of ephedrine in herbal mixtures using the optimized sensor and the recovery of spiked standard concentrations

Herbal product	EPH concentration in the sample (ppm ± % RSD)^a^	Spiked EPH standard (recovery% ± % RSD)^a^
Royal Regime Tea®	Nil	105.03 ± 2.41
Herbal mixture of unknown composition purchased from a local apothecary	227.01 ± 3.418	98.85 ± 4.81

a Average of five determination.

The accuracy of the proposed method was further assessed through the back determination of EPH spiked to the herbal products. Good recoveries and % RSD values were obtained as shown in (Table 7).

The developed method directly determines EPH in the traditional cup of tea; thus, reserves time, resources, and chemicals and releases fewer chemical wastes to the environment compared to the reported methods. The swift sensor responses enable high throughput analysis for a large number of samples within a relatively short time. The high efficiency of the sensor-enabled for the analysis of OTC herbal medications administered by patients suffering from sympathomimetic side effects and assists physicians to discover the etiology for such unexpected complications.

The developed potentiometric method considers environmental sustainability. The method can directly determine EPH in herbal tea products without sample preparation or derivatization, produces fewer wastes, and consumes no organic solvents and minimal chemicals reagents per analysis to fulfill the green analytical chemistry requirements.

The study compares the performance of the developed sensor to that of the reported inner liquid membrane sensors (Table 8). The sensor demonstrated a comparable slope, wider linear range, and a lower detection limit than most of the reported sensors (except for method VI^16^). Exclusively, the sensor had the widest pH working range and was the only sensor designed for the determination of EPH in herbal samples.

**Table:** Comparison between the proposed potentiometric method and reported electrochemical methods for ephedrine assay

Parameter	Proposed method	Reported electrochemical liquid membrane methods					
		Method I^14^	Method II^17^	Method III^20^	Method IV^19^	Method V^18^	Method VI^16^
Linear range (M)	1.00 × 10^−2^ to 6.00 × 10^−6^	1.00 × 10^−2^ to 1.00 × 10^−5^	1.00 × 10^−2^ to 5.00 × 10^−5^	8.00 × 10^−3^ to 3.00 × 10^−5^	1.00 × 10^−1^ to 2.00 × 10^−5^	1.00 × 10^−2^ to 5.00 × 10^−5^	1.00 × 10^−1^ to 2.00 × 10^−6^
Slope	58.86	55.00	58.20	57.00	Nernstian	55.79	56.00
LOD (M)	3.60 × 10^−6^	4.50 × 10^−6^	1.00 × 10^−4.5^	6.20 × 10^−6^	—	—	1.60 × 10^−6^
pH range	2–10	4–7	—	4–9	2–8	4–10	4–9
Sample	Herbal products	Pharmaceutical preparations	Pharmaceutical preparations	Pharmaceuticals and biological fluid	Pharmaceutical preparations	Pharmaceutical preparations	Pharmaceutical preparations

## 4. Conclusion

A green potentiometric method was developed for the analysis of ephedrine in pure form and herbal remedies. The method depends on a solid-state sensor composed of a calix[8]arene based membrane drop casted over a glassy carbon electrode previously coated with a polyaniline layer. The developed sensor has remarkable advantages being sustainable, sensitive, highly selective, and stable over a wide pH range (2–10). Besides, it responds rapidly to the change in EPH concentrations over a relatively long lifetime. These unique features encourage analysts to utilize the sensor for environmentally benign high throughput assay of EPH in a large number of samples within a short time. The developed sensor guides physicians in following up on the side effects suffered by patients consuming herbal supplements containing EPH on a regular basis.

## Conflicts of interest

Authors declare the absence of conflict of interest.

## Supplementary Material

RA-011-D0RA10254G-s001

## References

[cit1] Stunkard A. (2002). Int. J. Obes..

[cit2] Boozer C. N., Daly P. A., Homel P., Solomon J. L., Blanchard D., Nasser J. A., Strauss R., Meredith T. (2002). Int. J. Obes..

[cit3] Chan K. and Lin T. X., in Side Effects of Drugs Annual, Elsevier, 2009, vol. 31, pp. 745–756

[cit4] Brunton L. L. , Goodman & Gilman's the Pharmacological Basis of Therapeutics, McGraw-Hill, California, USA, 12th edn, 2011

[cit5] Pellati F., Benvenuti S. (2008). J. Pharm. Biomed. Anal..

[cit6] Roman M. C., Gray D., Luo G., McClanahan R., Perez R., Roper C., Roscoe V., Shevchuk C., Suen E., Sullivan D., Walther H. J. (2004). J. AOAC Int..

[cit7] Okamura N., Miki H., Harada T., Yamashita S., Masaoka Y., Nakamoto Y., Tsuguma M., Yoshitomi H., Yagi A. (1999). J. Pharm. Biomed. Anal..

[cit8] Niemann R. A., Gay M. L. (2003). J. Agric. Food Chem..

[cit9] Li H.-X., Ding M.-Y., Lv K., Yu J.-Y. (2002). J. Liq. Chromatogr. Relat. Technol..

[cit10] Ichikawa M., Udayama M., Imamura K., Shiraishi S., Matsuura H. (2003). Chem. Pharm. Bull..

[cit11] Dong Y.-M., An Q., Lu N.-W., Li N. (2015). Acta Chromatogr..

[cit12] Li H.-X., Ding M.-Y., Lv K., Yu J.-Y. (2001). J. Chromatogr. Sci..

[cit13] Liu Y.-M., Sheu S.-J. (1993). J. Chromatogr. A.

[cit14] Hassan S. S. M., Saoudi M. M. (1986). Analyst.

[cit15] Hassan S. S. M., Rechnitz G. A. (1986). Anal. Chem..

[cit16] Zareh M. M., El-Shiekh R., El-Bahnasawy R., Abo-El-Naga D. O. (1998). Microchem. J..

[cit17] Chamorro P. R., carmelo Diaz R. (1993). Talanta.

[cit18] Chamorro P. R., Diaz R. C. (1992). Analyst.

[cit19] Zareh M. M., Issa Y. M., Shoukry A. F., Shohaib R. E. (1993). J. Chem. Technol. Biotechnol..

[cit20] Hassan S. S. M., Kamel A. H., El-Naby H. A. (2013). Talanta.

[cit21] Saad A. S., Essam H. M. (2019). Electroanalysis.

[cit22] Draz M. E., Darwish H. W., Darwish I. A., Saad A. S. (2020). Food Chem..

[cit23] Buck R. P., Lindner E. (1994). Pure Appl. Chem..

[cit24] International Conference on Harmonization , ICH Harmonised Tripartite Guideline, Validation of analytical procedure: text and methodology, Q2 (R1), International Conference on Harmonization, Geneva, switzerland, 2005

[cit25] Umezawa Y., Bühlmann P., Umezawa K., Tohda K., Amemiya S. (2000). Pure Appl. Chem..

[cit26] Baumann E. W. (1968). Anal. Chim. Acta.

[cit27] Saad A. S., Edrees F. H., Elsaady M. T., Amin N. H., Abdelwahab N. S. (2020). J. Electrochem. Soc..

[cit28] Solsky R. L. (1990). Anal. Chem..

[cit29] Horvai G., Graf E., Toth K., Pungor E., Buck R. P. (1986). Anal. Chem..

[cit30] Toth K., Graf E., Horvai G., Pungor E., Buck R. P. (1986). Anal. Chem..

[cit31] Galal M. M., Saad A. S. (2020). RSC Adv..

[cit32] Beale J. M. , Block J. and Hill R., Organic medicinal and pharmaceutical chemistry, Wolters Kluwer, Lippincott Williams & Wilkins, London, UK, 12th edn, 2010

[cit33] Zareh M. M. , Plasticizers and their role in membrane selective electrodes, Shanghai, 2012

[cit34] Mihali C., Vaum N. (2012). Recent Adv. Plast..

[cit35] Ortuño J. A. , Tomás-Alonso F. and Rubio A. M., in Ionic Liquids in Separation Technology, Elsevier, 2014, pp. 275–299

[cit36] Bakker E. , in Encyclopedia of Analytical Sciences, 2nd edn, Elsevier, 2005, pp. 509–514

[cit37] O'Connor K. M., Arrigan D. W. M., Svehla G. (1995). Electroanalysis.

[cit38] Saad A. S., Naguib I. A., Draz M. E., Zaazaa H. E., Lashien A. S. (2018). J. Electrochem. Soc..

