# Supplementary material for: Calixarene based portable sensor for the direct assay of indiscriminate ephedrine content of weight loss herbal preparations
Source: RSC Adv. 2021 Apr 6;11(21):12833–44. doi: 10.1039/d0ra10254g (PMC8697355; doi:10.1039/d0ra10254g)
Supplement: RA-011-D0RA10254G-s001 [file RA-011-D0RA10254G-s001.pdf]

Electronic Supplementary Material (ESI) for RSC Advances.
This journal is © The Royal Society of Chemistry 2021

# Supplementary information

**Supplementary information S1.** IUPAC recommendations to calculate the sensor performance parameters:

## 1. Linear range:

The linear range of the electrode is defined as that part of the calibration curve through which a linear regression demonstrates no deviation from linearity by more than 2 mV.

## 2. Limit of detection (LOD):

The concentration of the primary ion at the point of intersection of the extrapolated lines of the Nernstian (high concentration) and nonresponsive (low concentration) segments of the calibration curve can be considered as an "attainable" detection limit under the stated experimental conditions.

## 3. Dynamic response time:

Practical response time required for the selected sensors to reach a value within ± 1 mV of the final stabilized potential upon increasing the concentration of EPH.

## 4. Reversibility:

The equilibrium potential was alternatively measured in $1.56\times10^{-4}$ and $1.57\times10^{-3}$ M ephedrine solutions.

**Supplementary information S2.** Derivation of the standard addition equation (equation 2).

Equation 2 was derived from Nernst equation as following:

- The measured potential in the sample solution of unknown concentration $C_u$ and known volume $V_s$.

$$E_1 = K + s\log C_s$$

Equation 1

Where (K) is the intercept, (s) is the slope and ($C_s$) is the sample molar concentration.

- The measured potential in the sample solution after addition of known volume $V_{st}$ of standard EPH solution of molar concentration $C_{st}$.

$$E_2 = K + s\log \frac{C_s V_s + C_{st} V_{st}}{V_t}$$

Equation 2

- $V_t$ is the total volume of the sample ($V_s$) and the added standard ($V_{st}$) solutions and $s$ is the slope measured in the blank matrix.

$$\Delta E = E_2 - E_1 = s\ log \frac{C_s V_s + C_{st} V_{st}}{C_s V_t}$$

Equation 3

$$10^{\left(\Delta E/S\right)} = \frac{C_u V_s + C_{st} V_{st}}{C_s V_t}$$

Equation 4

$$[C_s V_t \times 10^{\left(\Delta E/S\right)}] - C_s V_s = C_{st} V_{st}$$

Equation 5

$$C_s \left[V_t \times 10^{\left(\Delta E/S\right)} - V_s\right] = C_{st} V_{st}$$

Equation 6

$$C_s(M) = \frac{C_{st}V_{st}}{\left[V_t \times 10^{\left(\Delta E/S\right)} - V_s\right]}$$

Equation 7

$$C_s(g/L) = \frac{C_{st}V_{st}}{\left[V_t \times 10^{\left(\Delta E/S\right)} - V_s\right]} \times Mwt$$

Equation 8

$$C_s(g/g) = \frac{C_{st}V_{st}}{\left[V_t \times 10^{\left(\Delta E/S\right)} - V_s\right]} \times \frac{Mwt}{w} \times \frac{200}{1000}$$

Equation 9

$$C_s(ppm) = \frac{C_{st}V_{st}}{\left[V_t \times 10^{\left(\Delta E/S\right)} - V_s\right]} \times \frac{Mwt \times 200 \times 10^6}{1000 \times w}$$

Equation 10

For Ephedrine:

$$\frac{3.3048 \times 10^7 \times C_{st}V_{st}}{W \times \left[\left(V_t \times 10^{\left(\frac{\Delta E}{s}\right)}\right) - V_s\right]}$$

Equation 11

**Supplementary information S3. Post hoc statistical analysis within levels of statistically significant factors.**

**S3a.** Post hoc statistical analysis *P* values among the levels of the ion exchanger.

| Response | | Ion Exchanger (*P* value) | | | |
|---|---|---|---|---|---|
| | | AR | PM | PT | TPB |
| Slope | AR | | | | |
| | PM | **<u>0.0312</u>** | | | |
| | PT | 0.5527 | **<u>0.0237</u>** | | |
| | TPB | **<u>0.0483</u>** | 0.8841 | **<u>0.0015</u>** | |
| r | AR | | | | |
| | PM | 0.8741 | | | |
| | PT | 0.8810 | 0.8109 | | |
| | TPB | **<u>0.0431</u>** | **<u>0.0343</u>** | **<u>0.0029</u>** | |

**Table S3b.** Post hoc statistical analysis *P* values among the levels of the plasticizer.

| Response | | Plasticizer (*P* value) | | | | |
|---|---|---|---|---|---|---|
| | | DOP | NPOE | DBP | NPPE | TCP |
| Slope | DOP | | | | | |
| | NPOE | 0.3347 | | | | |
| | DBP | **0.0322** | **<0.0001** | | | |
| | NPPE | 0.5144 | 0.6126 | **0.0004** | | |
| | TCP | **<0.0001** | **<0.0001** | **0.0091** | **<0.0001** | |
| LOQ | DOP | | | | | |
| | NPOE | 0.9152 | | | | |
| | DBP | **<0.0001** | **<0.001** | | | |
| | NPPE | 0.9211 | 0.9741 | **<0.0001** | | |
| | TCP | 0.9887 | 0.9847 | **<0.0001** | 0.9844 | |
| r | DOP | | | | | |
| | NPOE | **<0.0001** | | | | |
| | DBP | 0.0518 | **<0.0001** | | | |
| | NPPE | 0.9120 | **<0.0001** | 0.1019 | | |
| | TCP | 0.9434 | **<0.0001** | **0.0371** | 0.9270 | |

**Table S3c.** Post hoc statistical analysis *P* values among the levels of the ionophore.

| Response | | Ionophore (*P* value) | | | | |
|---|---|---|---|---|---|---|
| | | CX-4 | CX-6 | CX-8 | β-CD | β-CD/CX-8 |
| Slope | CX-4 | | | | | |
| | CX-6 | **<u><0.0001</u>** | | | | |
| | CX-8 | **<u><0.0001</u>** | 0.6071 | | | |
| | β-CD | **<u><0.0001</u>** | 0.6275 | 0.9118 | | |
| | β-CD/CX-8 | **<u><0.0001</u>** | 0.8379 | 0.8818 | 0.7825 | |
| LOQ | CX-4 | | | | | |
| | CX-6 | **<u><0.0001</u>** | | | | |
| | CX-8 | **<u><0.0001</u>** | 0.9795 | | | |
| | β-CD | **<u><0.0001</u>** | 0.9804 | 0.9812 | | |
| | β-CD/CX-8 | **<u><0.0001</u>** | 0.9734 | 0.9776 | 0.9800 | |
| r | CX-4 | | | | | |
| | CX-6 | 0.9183 | | | | |
| | CX-8 | **<u><0.0001</u>** | **<u><0.0001</u>** | | | |
| | β-CD | 0.1073 | 0.0847 | **<u>0.0271</u>** | | |
| | β-CD/CX-8 | **<u><0.0001</u>** | **<u><0.0001</u>** | 0.7414 | **<u>0.0440</u>** | |

**Table S3d.** Post hoc statistical analysis *P* values among the levels of the conditioning solution concentration.

| Response | | Conditioning solution Concentration (*P* value) | | |
|---|---|---|---|---|
| | | 0.01 | 0.001 | 0.0001 |
| Slope | 0.01 | | | |
| | 0.001 | **<u><0.0001</u>** | | |
| | 0.0001 | **<u><0.0001</u>** | **<u><0.0001</u>** | |
| LOQ | 0.01 | | | |
| | 0.001 | **<u><0.0001</u>** | | |
| | 0.0001 | **<u><0.0001</u>** | **<u><0.0001</u>** | |
| r | 0.01 | | | |
| | 0.001 | **<u><0.0001</u>** | | |
| | 0.0001 | **<u><0.0001</u>** | **<u><0.0001</u>** | |

**Table S3e.** Post hoc statistical analysis *P* values among the levels of the membrane thickness.

| Response | | Membrane Thickness (*P* value) | | |
|---|---|---|---|---|
| | | 0.12 | 0.1 | 0.08 |
| Slope | 0.12 | | | |
| | 0.1 | **<u>≤0.0001</u>** | | |
| | 0.08 | **<u>≤0.0001</u>** | **<u>≤0.0001</u>** | |
| LOQ | 0.12 | | | |
| | 0.1 | **<u>≤0.0001</u>** | | |
| | 0.08 | **<u>≤0.0001</u>** | **<u>≤0.0001</u>** | |
| r | 0.12 | | | |
| | 0.1 | **<u>≤0.0001</u>** | | |
| | 0.08 | **<u>≤0.0001</u>** | **<u>≤0.0001</u>** | |
